# *N*-(Diphenyl­carbamothio­yl)-3-methyl­benzamide

**DOI:** 10.1107/S1600536809002554

**Published:** 2009-01-23

**Authors:** Gün Binzet, Ulrich Flörke, Nevzat Külcü, Hakan Arslan

**Affiliations:** a Department of Chemistry, Faculty of Arts and Science, Mersin University, Mersin, TR 33343, Turkey; b Department of Chemistry, University of Paderborn, Paderborn D 33098, Germany; c Department of Natural Sciences, Fayetteville State University, Fayetteville, NC 28301, USA; d Department of Chemistry, Faculty of Pharmacy, Mersin University, Mersin, TR 33169, Turkey

## Abstract

The synthesis of the title compound, C_21_H_18_N_2_OS, involves the reaction of 3-methyl­benzoyl chloride with potassium thio­cyanate in dry acetone followed by condensation of the 3-methyl­benzoyl isothio­cyanate with diphenyl­amine. The carbonyl [C—O = 1.215 (2) Å] and thio­carbonyl [C—S = 1.6721 (17) Å] distances indicate that these correspond to double bonds. The short C—N bonds at the center of the mol­ecule reveal the effects of resonance in this part of the mol­ecule. The conformation of the mol­ecule with respect to the thio­carbonyl and carbonyl groups is twisted. The 3-methyl­phenyl and two phenyl rings are also twisted, with dihedral angles of 75.67 (9) and 14.91 (9)°. The phenyl rings are rotated out of the mean plane of the N—C—S—N atoms by 66.87 (8) and 78.40 (9)°. Pairs of mol­ecules are linked into centrosymmetric dimers *via* inter­molecular N—H⋯S inter­actions and a C—H⋯O link also occurs. The dimers are stacked along the *a* axis.

## Related literature

For synthesis, see: Özer *et al.* (2009 ▶); Mansuroğlu *et al.* (2008 ▶); Uğur *et al.* (2006 ▶); Arslan *et al.* (2003*a* ▶, and references therein). For general background, see: Koch (2001 ▶); El Aamrani *et al.* (1998 ▶, 1999 ▶). For related compounds, see: Arslan *et al.* (2003*b* ▶,*c* ▶, 2004 ▶); Khawar Rauf *et al.* (2009*a* ▶,*b* ▶,*c* ▶,*d* ▶).
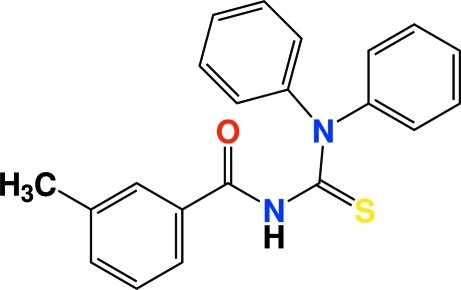


## Experimental

### 

#### Crystal data

C_21_H_18_N_2_OS
*M* *_r_* = 346.43
Orthorhombic, 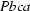

*a* = 10.6928 (12) Å
*b* = 16.7647 (17) Å
*c* = 19.865 (2) Å
*V* = 3561.0 (6) Å^3^
*Z* = 8
Mo *K*α radiation
μ = 0.19 mm^−1^
*T* = 120 (2) K
0.41 × 0.23 × 0.08 mm

#### Data collection

Bruker SMART APEX diffractometer
Absorption correction: multi-scan (*SADABS*; Bruker, 2002 ▶) *T*_min_ = 0.925, *T*_max_ = 0.985
29957 measured reflections
4241 independent reflections
2955 reflections with *I* > 2σ(*I*)
*R*_int_ = 0.098

#### Refinement

*R*[*F* ^2^ > 2σ(*F* ^2^)] = 0.047
*wR*(*F* ^2^) = 0.101
*S* = 0.95
4241 reflections
231 parameters
1 restraint
H atoms treated by a mixture of independent and constrained refinement
Δρ_max_ = 0.28 e Å^−3^
Δρ_min_ = −0.38 e Å^−3^

### 

Data collection: *SMART* (Bruker, 2002 ▶); cell refinement: *SAINT* (Bruker, 2002 ▶); data reduction: *SAINT*; program(s) used to solve structure: *SHELXTL* (Sheldrick, 2008 ▶); program(s) used to refine structure: *SHELXTL*; molecular graphics: *SHELXTL*; software used to prepare material for publication: *SHELXTL*.

## Supplementary Material

Crystal structure: contains datablocks I, global. DOI: 10.1107/S1600536809002554/at2712sup1.cif

Structure factors: contains datablocks I. DOI: 10.1107/S1600536809002554/at2712Isup2.hkl

Additional supplementary materials:  crystallographic information; 3D view; checkCIF report

## Figures and Tables

**Table 1:** Hydrogen-bond geometry (Å, °)

*D*—H⋯*A*	*D*—H	H⋯*A*	*D*⋯*A*	*D*—H⋯*A*
N1—H1⋯S1^i^	0.899 (14)	2.525 (14)	3.4123 (15)	169.3 (13)
C5—H5*B*⋯O1^ii^	0.98	2.59	3.461 (2)	148

Symmetry codes: (i) 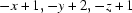; (ii) 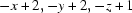.

**Fig. 1.**
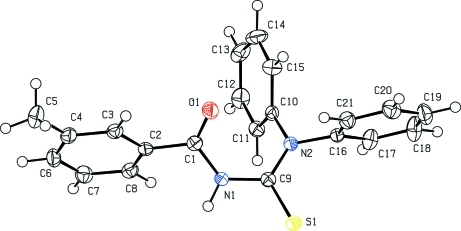
The molecular structure of (I). Displacement ellipsoids are drawn at the 50% probability level.

**Fig. 2.**
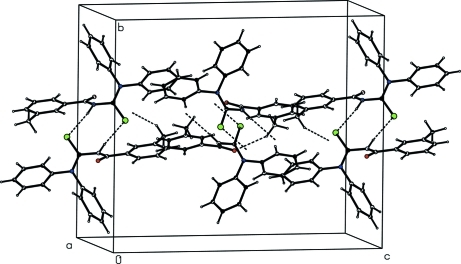
A packing diagram for (I). Hydrogen bonds are shown as dashed lines.

## supplementary crystallographic information

### Comment

Thioureas are basic and excellent ligands for the transition group metals, so many derivatives have been prepared for use in the liquid–liquid extraction and separation of some transition metal ions (Koch, 2001). Especially, *N*-benzoylthiourea derivatives have been successfully used in the extraction of copper(II) and gold(III) ions (El Aamrani *et al.*, 1998, 1999).

Recently, we have focused on the synthesis, characterization, crystal structure, thermal behavior and antimicrobial activity of new thiourea derivatives (Özer *et al.*, 2009; Mansuroğlu *et al.*, 2008; Uğur *et al.*, 2006; Arslan *et al.*, 2003*c*, and references therein). In the present study, we have synthesized and characterized a new thiourea derivative, *N*-(diphenylcarbamothioyl)-3-methylbenzamide, (I). The molecular structure of the title compound is depicted in Fig. 1.

The carbonyl (C1—O1 = 1.215 (2) Å) and thiocarbonyl (C9—S1 = 1.6721 (17) Å) distances indicates that these correspond to double bonds and are comparable to those observed for *N*'-(4-chlorobenzoyl)-*N*,*N*-diphenylthiourea [1.213 (3) Å for C—O and 1.664 (2) Å for C—S] (Arslan *et al.*, 2003*a*), 1-(4-chloro-benzoyl)-3-naphthalen-1-yl-thiourea [1.224 (2) Å for C—O and 1.6696 (17) Å for C—S] (Arslan *et al.*, 2003*b*), 1-(2-chloro-benzoyl)-3-*p*-tolyl-thiourea [1.216 (2) Å for C—O and 1.666 (2) Å for C—S] (Arslan *et al.*, 2004), 1-(4-chlorobenzoyl)-3-(2,4,6-trichlorophenyl)thiourea [1.229 (3) Å for C—O and 1.666 (2) Å for C—S] (Khawar Rauf *et al.*, 2009*b*).

The C—N bond distances [C1—N1 = 1.395 (2) Å, C9—N1 = 1.396 (2) Å and C9—N2 = 1.344 (2) Å] observed in the title compound are consistent with those reported for the other thiourea derivatives (1-(3-chlorophenyl)-3-(2,6-dichlorobenzoyl)thiourea (Khawar Rauf *et al.*, 2009*d*), 1-(3-chlorobenzoyl)-3-(2,3-dimethylphenyl)thiourea (Khawar Rauf *et al.*, 2009*c*), 1-(2,6-dichlorobenzoyl)-3-(2,3,5,6-tetrachlorophenyl)thiourea (Khawar Rauf *et al.*, 2009*a*), suggesting a partial double-bond character.

The conformation of the title molecule with respect to the thiocarbonyl and carbonyl moieties is twisted, as reflected by the C9—N1—C1—O1 and C1—N1—C9—N2 torsion angles of 0.1 (3)° and -56.1 (2)°, respectively. The 3-methylphenyl and phenyl rings (C10–C15 and C16–C21 rings) are also twisted with dihedral angles of 75.67 (9)° and 14.91 (9)°, respectively.

The atom N2 is *sp*^2^-hybridized, because of the sum of the angles around atom N2 is 359.91 (13)°. The phenyl rings are rotated out of the mean plane of the N1–C9–S1–N2 atoms by 66.87 (8)° (C10–C15 ring) and 78.40 (9)° (C16–C21 ring). In addition, the dihedral angle between C10–C15 ring and C16–C21 ring is 87.81 (9)°.

As can be seen from the packing diagram (Fig. 2), intermolecular N—H···S hydrogen bond (Table 1) links the molecules into dimers, which are stacked along the *a* axis. The C—H···O intermolecular contact is also listed in Table 1.

### Experimental

The title compound was prepared with a procedure similar to that reported in the literature (Arslan *et al.*, 2003*c*). A solution of 3-methylbenzoyl chloride (0.01 mol) in acetone (50 ml) was added dropwise to a suspension of potassium thiocyanate (0.01 mol) in acetone (30 ml). The reaction mixture was heated under reflux for 30 min, and then cooled to room temperature. A solution of diphenylamine (0.01 mol) in acetone (10 ml) was added and the resulting mixture was stirred for 2 h. Hydrochloric acid (0.1 N, 300 ml) was added to the solution, which was then filtered. The solid product was washed with water and purifed by recrystalization from an ethanol:dichloromethane mixture (1:2). Analysis calculated for C_21_H_18_N_2_OS: C 72.8, H 5.2, N  8.1%. Found: C 72.6, H 5.2, N 8.0%.

### Refinement

H atoms bound to C atoms were placed geometrically and allowed to ride during subsequent refinement with C—H = 0.95 and 0.98 Å and *U*_iso_(H) = 1.2 or 1.5 *U*_eq_(C). The nitrogen-bound H atom was located in a difference Fourier map and refined freely.

### Crystal data

**Table:**

C_21_H_18_N_2_OS	*F*(000) = 1456
*M**_r_* = 346.43	*D*_x_ = 1.292 Mg m^−^^3^
Orthorhombic, *P**b**c**a*	Mo *K*α radiation, λ = 0.71073 Å
Hall symbol: -P 2ac 2ab	Cell parameters from 801 reflections
*a* = 10.6928 (12) Å	θ = 2.5–25.9°
*b* = 16.7647 (17) Å	µ = 0.19 mm^−^^1^
*c* = 19.865 (2) Å	*T* = 120 K
*V* = 3561.0 (6) Å^3^	Prism, colourless
*Z* = 8	0.41 × 0.23 × 0.08 mm

### Data collection

**Table:**

Bruker SMART APEX diffractometer	4241 independent reflections
Radiation source: sealed tube	2955 reflections with *I* > 2σ(*I*)
graphite	*R*_int_ = 0.098
φ and ω scans	θ_max_ = 27.9°, θ_min_ = 2.1°
Absorption correction: multi-scan (*SADABS*; Bruker, 2002)	*h* = −14→14
*T*_min_ = 0.925, *T*_max_ = 0.985	*k* = −22→19
29957 measured reflections	*l* = −26→26

### Refinement

**Table:**

Refinement on *F*^2^	Primary atom site location: structure-invariant direct methods
Least-squares matrix: full	Secondary atom site location: difference Fourier map
*R*[*F*^2^ > 2σ(*F*^2^)] = 0.047	Hydrogen site location: difference Fourier map
*wR*(*F*^2^) = 0.101	H atoms treated by a mixture of independent and constrained refinement
*S* = 0.95	*w* = 1/[σ^2^(*F*_o_^2^) + (0.0429*P*)^2^] where *P* = (*F*_o_^2^ + 2*F*_c_^2^)/3
4241 reflections	(Δ/σ)_max_ = 0.001
231 parameters	Δρ_max_ = 0.28 e Å^−^^3^
1 restraint	Δρ_min_ = −0.38 e Å^−^^3^

### Special details

**Table:**

Geometry. All esds (except the esd in the dihedral angle between two l.s. planes) are estimated using the full covariance matrix. The cell esds are taken into account individually in the estimation of esds in distances, angles and torsion angles; correlations between esds in cell parameters are only used when they are defined by crystal symmetry. An approximate (isotropic) treatment of cell esds is used for estimating esds involving l.s. planes.
Refinement. Refinement of F^2^ against ALL reflections. The weighted R-factor wR and goodness of fit S are based on F^2^, conventional R-factors R are based on F, with F set to zero for negative F^2^. The threshold expression of F^2^ > 2sigma(F^2^) is used only for calculating R-factors(gt) etc. and is not relevant to the choice of reflections for refinement. R-factors based on F^2^ are statistically about twice as large as those based on F, and R- factors based on ALL data will be even larger.

### Fractional atomic coordinates and isotropic or equivalent isotropic displacement parameters (Å^2^)

**Table:**

	*x*	*y*	*z*	*U*_iso_*/*U*_eq_	
S1	0.49155 (4)	0.96594 (3)	0.60687 (2)	0.02357 (13)	
O1	0.83581 (11)	0.88560 (8)	0.53805 (6)	0.0282 (3)	
N1	0.63123 (14)	0.90441 (8)	0.50788 (7)	0.0194 (3)	
H1	0.5892 (15)	0.9383 (8)	0.4811 (7)	0.029 (5)*	
N2	0.61147 (13)	0.82693 (8)	0.60385 (7)	0.0198 (3)	
C1	0.75893 (17)	0.89869 (10)	0.49441 (8)	0.0204 (4)	
C2	0.79282 (16)	0.91117 (10)	0.42236 (8)	0.0201 (4)	
C3	0.90683 (17)	0.94747 (10)	0.40766 (9)	0.0226 (4)	
H3A	0.9603	0.9633	0.4434	0.027*	
C4	0.94370 (18)	0.96101 (10)	0.34132 (9)	0.0252 (4)	
C5	1.0663 (2)	1.00079 (12)	0.32634 (10)	0.0360 (5)	
H5A	1.1349	0.9638	0.3362	0.054*	
H5B	1.0750	1.0486	0.3543	0.054*	
H5C	1.0692	1.0159	0.2787	0.054*	
C6	0.86364 (18)	0.93615 (11)	0.29006 (9)	0.0283 (4)	
H6A	0.8863	0.9456	0.2445	0.034*	
C7	0.75192 (18)	0.89805 (11)	0.30421 (8)	0.0280 (4)	
H7A	0.7001	0.8801	0.2685	0.034*	
C8	0.71531 (17)	0.88605 (10)	0.37039 (9)	0.0237 (4)	
H8A	0.6378	0.8608	0.3801	0.028*	
C9	0.58249 (16)	0.89564 (10)	0.57267 (8)	0.0190 (4)	
C10	0.65779 (16)	0.75696 (10)	0.56929 (8)	0.0200 (4)	
C11	0.58848 (18)	0.72236 (10)	0.51815 (8)	0.0241 (4)	
H11A	0.5118	0.7455	0.5040	0.029*	
C12	0.63308 (19)	0.65307 (10)	0.48777 (9)	0.0307 (5)	
H12A	0.5872	0.6291	0.4522	0.037*	
C13	0.7437 (2)	0.61921 (11)	0.50926 (10)	0.0346 (5)	
H13A	0.7741	0.5723	0.4881	0.042*	
C14	0.81025 (19)	0.65320 (11)	0.56141 (11)	0.0360 (5)	
H14A	0.8852	0.6289	0.5768	0.043*	
C15	0.76792 (18)	0.72271 (11)	0.59126 (9)	0.0287 (4)	
H15A	0.8143	0.7467	0.6267	0.034*	
C16	0.59063 (16)	0.81692 (10)	0.67524 (8)	0.0191 (4)	
C17	0.50108 (18)	0.76351 (11)	0.69656 (9)	0.0285 (4)	
H17A	0.4504	0.7359	0.6649	0.034*	
C18	0.4862 (2)	0.75071 (12)	0.76492 (9)	0.0371 (5)	
H18A	0.4246	0.7143	0.7803	0.045*	
C19	0.5600 (2)	0.79033 (11)	0.81074 (9)	0.0342 (5)	
H19A	0.5486	0.7814	0.8576	0.041*	
C20	0.65082 (19)	0.84305 (12)	0.78887 (9)	0.0317 (5)	
H20A	0.7026	0.8697	0.8206	0.038*	
C21	0.66613 (18)	0.85692 (11)	0.72048 (9)	0.0258 (4)	
H21A	0.7277	0.8934	0.7050	0.031*	

### Atomic displacement parameters (Å^2^)

**Table:**

	*U*^11^	*U*^22^	*U*^33^	*U*^12^	*U*^13^	*U*^23^
S1	0.0282 (3)	0.0204 (2)	0.0221 (2)	0.00679 (19)	0.0028 (2)	0.00050 (19)
O1	0.0228 (7)	0.0371 (8)	0.0248 (7)	−0.0013 (6)	−0.0014 (6)	0.0050 (6)
N1	0.0203 (8)	0.0184 (8)	0.0197 (7)	0.0027 (6)	0.0013 (6)	0.0037 (6)
N2	0.0226 (8)	0.0181 (7)	0.0186 (7)	0.0038 (6)	0.0025 (6)	0.0010 (6)
C1	0.0207 (9)	0.0157 (9)	0.0246 (9)	−0.0010 (7)	0.0012 (8)	−0.0003 (7)
C2	0.0237 (10)	0.0155 (9)	0.0210 (9)	0.0059 (7)	0.0022 (7)	0.0008 (7)
C3	0.0225 (10)	0.0206 (9)	0.0248 (9)	0.0027 (8)	0.0010 (8)	−0.0007 (7)
C4	0.0290 (10)	0.0188 (9)	0.0278 (10)	0.0045 (8)	0.0068 (8)	0.0008 (8)
C5	0.0366 (12)	0.0390 (12)	0.0324 (11)	−0.0055 (10)	0.0126 (9)	−0.0003 (9)
C6	0.0384 (12)	0.0260 (10)	0.0204 (9)	0.0050 (9)	0.0079 (8)	0.0023 (8)
C7	0.0320 (11)	0.0291 (10)	0.0231 (9)	0.0031 (9)	−0.0026 (8)	−0.0032 (8)
C8	0.0221 (10)	0.0218 (10)	0.0271 (9)	0.0018 (8)	0.0018 (8)	−0.0010 (8)
C9	0.0177 (9)	0.0196 (9)	0.0196 (9)	−0.0018 (7)	−0.0022 (7)	−0.0003 (7)
C10	0.0228 (10)	0.0163 (9)	0.0208 (9)	0.0013 (7)	0.0055 (7)	0.0032 (7)
C11	0.0263 (11)	0.0214 (9)	0.0247 (9)	0.0010 (8)	0.0013 (8)	0.0025 (7)
C12	0.0389 (12)	0.0223 (10)	0.0310 (10)	−0.0028 (9)	0.0046 (9)	−0.0046 (8)
C13	0.0370 (12)	0.0186 (10)	0.0482 (12)	0.0033 (9)	0.0164 (10)	−0.0037 (9)
C14	0.0250 (11)	0.0288 (11)	0.0542 (13)	0.0104 (9)	0.0037 (10)	0.0050 (10)
C15	0.0264 (11)	0.0275 (11)	0.0322 (10)	0.0034 (8)	−0.0042 (8)	0.0007 (8)
C16	0.0222 (10)	0.0173 (8)	0.0178 (8)	0.0045 (7)	0.0025 (7)	0.0013 (7)
C17	0.0304 (11)	0.0289 (10)	0.0263 (10)	−0.0072 (9)	0.0060 (8)	−0.0042 (8)
C18	0.0515 (14)	0.0301 (11)	0.0297 (11)	−0.0106 (10)	0.0151 (10)	−0.0013 (9)
C19	0.0529 (14)	0.0291 (11)	0.0206 (10)	0.0054 (10)	0.0078 (9)	0.0029 (8)
C20	0.0378 (13)	0.0328 (11)	0.0243 (10)	0.0024 (9)	−0.0047 (9)	−0.0039 (8)
C21	0.0256 (11)	0.0259 (10)	0.0259 (9)	−0.0025 (8)	0.0002 (8)	0.0013 (8)

### Geometric parameters (Å, °)

**Table:**

S1—C9	1.6721 (17)	C10—C15	1.381 (2)
O1—C1	1.215 (2)	C10—C11	1.385 (2)
N1—C1	1.395 (2)	C11—C12	1.393 (2)
N1—C9	1.396 (2)	C11—H11A	0.9500
N1—H1	0.899 (14)	C12—C13	1.379 (3)
N2—C9	1.344 (2)	C12—H12A	0.9500
N2—C16	1.445 (2)	C13—C14	1.380 (3)
N2—C10	1.447 (2)	C13—H13A	0.9500
C1—C2	1.491 (2)	C14—C15	1.384 (3)
C2—C8	1.389 (2)	C14—H14A	0.9500
C2—C3	1.393 (2)	C15—H15A	0.9500
C3—C4	1.394 (2)	C16—C17	1.378 (2)
C3—H3A	0.9500	C16—C21	1.382 (2)
C4—C6	1.394 (3)	C17—C18	1.384 (2)
C4—C5	1.501 (3)	C17—H17A	0.9500
C5—H5A	0.9800	C18—C19	1.375 (3)
C5—H5B	0.9800	C18—H18A	0.9500
C5—H5C	0.9800	C19—C20	1.383 (3)
C6—C7	1.383 (3)	C19—H19A	0.9500
C6—H6A	0.9500	C20—C21	1.388 (2)
C7—C8	1.386 (2)	C20—H20A	0.9500
C7—H7A	0.9500	C21—H21A	0.9500
C8—H8A	0.9500		
			
C1—N1—C9	122.34 (14)	C15—C10—C11	120.93 (16)
C1—N1—H1	114.8 (12)	C15—C10—N2	118.63 (15)
C9—N1—H1	115.1 (12)	C11—C10—N2	120.30 (15)
C9—N2—C16	121.07 (14)	C10—C11—C12	118.94 (18)
C9—N2—C10	123.73 (14)	C10—C11—H11A	120.5
C16—N2—C10	115.11 (13)	C12—C11—H11A	120.5
O1—C1—N1	122.56 (16)	C13—C12—C11	120.17 (18)
O1—C1—C2	123.08 (16)	C13—C12—H12A	119.9
N1—C1—C2	114.35 (15)	C11—C12—H12A	119.9
C8—C2—C3	119.91 (16)	C12—C13—C14	120.30 (18)
C8—C2—C1	121.72 (16)	C12—C13—H13A	119.9
C3—C2—C1	118.36 (16)	C14—C13—H13A	119.9
C2—C3—C4	121.10 (17)	C13—C14—C15	120.05 (19)
C2—C3—H3A	119.5	C13—C14—H14A	120.0
C4—C3—H3A	119.5	C15—C14—H14A	120.0
C6—C4—C3	117.92 (17)	C10—C15—C14	119.59 (18)
C6—C4—C5	121.64 (17)	C10—C15—H15A	120.2
C3—C4—C5	120.44 (17)	C14—C15—H15A	120.2
C4—C5—H5A	109.5	C17—C16—C21	121.43 (16)
C4—C5—H5B	109.5	C17—C16—N2	118.97 (15)
H5A—C5—H5B	109.5	C21—C16—N2	119.46 (16)
C4—C5—H5C	109.5	C16—C17—C18	118.82 (18)
H5A—C5—H5C	109.5	C16—C17—H17A	120.6
H5B—C5—H5C	109.5	C18—C17—H17A	120.6
C7—C6—C4	121.31 (17)	C19—C18—C17	120.57 (18)
C7—C6—H6A	119.3	C19—C18—H18A	119.7
C4—C6—H6A	119.3	C17—C18—H18A	119.7
C6—C7—C8	120.24 (17)	C18—C19—C20	120.24 (17)
C6—C7—H7A	119.9	C18—C19—H19A	119.9
C8—C7—H7A	119.9	C20—C19—H19A	119.9
C7—C8—C2	119.48 (17)	C19—C20—C21	119.82 (18)
C7—C8—H8A	120.3	C19—C20—H20A	120.1
C2—C8—H8A	120.3	C21—C20—H20A	120.1
N2—C9—N1	115.40 (15)	C16—C21—C20	119.11 (18)
N2—C9—S1	123.43 (13)	C16—C21—H21A	120.4
N1—C9—S1	121.15 (12)	C20—C21—H21A	120.4
			
C9—N1—C1—O1	0.1 (2)	C16—N2—C10—C15	−57.2 (2)
C9—N1—C1—C2	−179.01 (15)	C9—N2—C10—C11	−57.8 (2)
O1—C1—C2—C8	146.21 (18)	C16—N2—C10—C11	118.63 (17)
N1—C1—C2—C8	−34.7 (2)	C15—C10—C11—C12	−1.6 (3)
O1—C1—C2—C3	−32.3 (2)	N2—C10—C11—C12	−177.31 (15)
N1—C1—C2—C3	146.80 (15)	C10—C11—C12—C13	0.9 (3)
C8—C2—C3—C4	1.6 (3)	C11—C12—C13—C14	0.7 (3)
C1—C2—C3—C4	−179.81 (15)	C12—C13—C14—C15	−1.7 (3)
C2—C3—C4—C6	−0.8 (3)	C11—C10—C15—C14	0.6 (3)
C2—C3—C4—C5	179.50 (16)	N2—C10—C15—C14	176.38 (16)
C3—C4—C6—C7	−1.0 (3)	C13—C14—C15—C10	1.1 (3)
C5—C4—C6—C7	178.66 (17)	C9—N2—C16—C17	112.65 (19)
C4—C6—C7—C8	2.1 (3)	C10—N2—C16—C17	−63.9 (2)
C6—C7—C8—C2	−1.2 (3)	C9—N2—C16—C21	−71.6 (2)
C3—C2—C8—C7	−0.6 (3)	C10—N2—C16—C21	111.85 (18)
C1—C2—C8—C7	−179.09 (16)	C21—C16—C17—C18	0.7 (3)
C16—N2—C9—N1	166.64 (14)	N2—C16—C17—C18	176.37 (16)
C10—N2—C9—N1	−17.1 (2)	C16—C17—C18—C19	−0.3 (3)
C16—N2—C9—S1	−14.9 (2)	C17—C18—C19—C20	−0.5 (3)
C10—N2—C9—S1	161.33 (13)	C18—C19—C20—C21	1.0 (3)
C1—N1—C9—N2	−56.1 (2)	C17—C16—C21—C20	−0.2 (3)
C1—N1—C9—S1	125.41 (15)	N2—C16—C21—C20	−175.88 (16)
C9—N2—C10—C15	126.34 (18)	C19—C20—C21—C16	−0.6 (3)

### Hydrogen-bond geometry (Å, °)

**Table:**

*D*—H···*A*	*D*—H	H···*A*	*D*···*A*	*D*—H···*A*
N1—H1···S1^i^	0.90 (1)	2.53 (1)	3.4123 (15)	169 (1)
C5—H5B···O1^ii^	0.98	2.59	3.461 (2)	148

Symmetry codes: (i) −*x*+1, −*y*+2, −*z*+1; (ii) −*x*+2, −*y*+2, −*z*+1.
